# Analysis of virulence factors in extracellular vesicles secreted by *Naegleria fowleri*

**DOI:** 10.1007/s00436-024-08378-9

**Published:** 2024-10-21

**Authors:** Itzel Berenice Rodríguez-Mera, Saúl Rojas-Hernández, Karla Alejandra Barrón-Graciano, María Maricela Carrasco-Yépez

**Affiliations:** 1 Universidad Nacional Autónoma de México, Grupo CyMA, UIICSE, FES Iztacala, Laboratorio de Microbiología Ambiental, Estado de México, Tlalnepantla de Baz, Mexico; 2 Laboratorio de Inmunología Molecular y de Mucosas, Instituto Politécnico Nacional, Escuela Superior de Medicina, Mexico City, Mexico

**Keywords:** *Naegleria fowleri*, Extracellular vesicles, Pathogenicity mechanism, Virulence factors, Biomarkers

## Abstract

*Naegleria fowleri* is the etiological agent of primary amebic meningoencephalitis (PAM), a rapidly progressive acute and fulminant infection that affects the central nervous system, particularly of children and young adults, which has a mortality rate greater than 95%, and its symptomatologic similarity with other meningitis caused by virus or bacteria makes it difficult to make a quick and timely diagnosis that prevents the progression of the infection. It is necessary to know the antigenic determinants as well as the pathogenicity mechanisms of this amoeba to implement strategies that allow for better antiamoebic therapeutic and diagnostic targets that directly impact the health sector. Therefore, the aim of this work was to analyze some virulence factors as part of extracellular vesicle (EV) cargo secreted by *N. fowleri*. The EV secretion to the extracellular medium was evaluated in trophozoites fixed and incubated with anti-*N. fowleri* antibody while molecular identification of EV cargo was performed by SDS-PAGE, Western blot, and RT-PCR. Our results showed that *N. fowleri* secretes a wide variety of vesicle sizes ranging from 0.2 to > 2 μm, and these EVs were recognized by antibodies anti-Naegleropore B, anti-19 kDa polypeptide band, anti-membrane protein Mp2CL5, anti-protease cathepsin B, and anti-actin. Furthermore, these vesicles were localized in the trophozoites cytoplasm or secreted into the extracellular medium. Specifically in relation to small vesicles, our purified exosomes were recognized by CD63 and Hsp70 markers, along with the previously mentioned proteins. RT-PCR analysis was made through the isolation of EVs from *N. fowleri* trophozoite culture by concentration, filtration, and ultracentrifugation. Interestingly, we obtained PCR products for Nfa1, NPB, Mp2CL5, and CatB genes as part of exosomes cargo. This suggests that the molecules identified in this work could play an important role in communication as well as in infectious processes caused by this amoeba. Therefore, the study and characterization of the pathogenicity mechanisms, as well as the virulence factors released by *N. fowleri* remains a key point to provide valuable information for the development of therapeutic treatments, vaccine design, or biomarkers for a timely diagnosis against infections caused by protozoa.

**Supplementary Information:**

The online version contains supplementary material available at 10.1007/s00436-024-08378-9.

## Introduction

*Naegleria fowleri* Carter, 1970 is the etiological agent of primary amoebic meningoencephalitis (PAM), an acute, fulminant, rapidly progressive infection that affects the central nervous system of children and young adults who have engaged in aquatic activities in natural or artificial freshwater bodies of water. This infection has a mortality rate greater than 95% and a progression period of 7–12 days (Dereeper et al. 2022; Nadeem et al. 2023).

Previously, *N. fowleri* antigens were detected and identified through protein analysis, which were suggested to be involved in the protection mediated by antibodies of mice immunized with total extract plus cholera toxin and challenged with the lethal dose of *N. fowleri* trophozoites. The identification of these antigens included polypeptide bands of 250, 100, 70, 50, 37, and 19 kDa (the latter being one of the most antigenic). When these polypeptide bands were analyzed by mass spectrometry, we found matched peptides for fragments of membrane proteins, actin, heat shock protein 70 (Hsp70), among others (Rojas-Hernández et al. 2020). Interestingly, these proteins have been considered important virulence factors because they are overexpressed in more virulent strains compared to less virulent or non-pathogenic strains. Thus, they have been considered to play a significant role in the cytopathic effect, as well as in the invasion and migration of *N. fowleri* trophozoites (Flores-Suárez et al. 2024; Gutiérrez-Sánchez et al. 2020; Jamerson et al. 2017; Réveiller et al. 2001; Sohn et al. 2019; Zysset-Burri et al. 2014). We understand that pathogenicity is a complex process that depends mainly on the virulence of the strain and the size of the inoculum (Nadeem et al. 2023). Although different molecules have been reported that may participate in the progress of the infection caused by *N. fowleri*, the pathogenicity mechanisms are still not clear. A wide variety of proteins have been linked to the ability of *N. fowleri* to cause damage; these include membrane proteins such as Mp2CL5 and Nfa1, proteases, and saposin-like proteins like pore-forming peptides A and B (Güémez and García, 2021; Marciano-Cabral and Cabral 2007).

On the other hand, the mechanisms by which virulence factor are released remain unclear. Recent reviews indicate that EVs released by various pathogenic microorganisms can contain virulence factors responsible for extensive damage of host tissue and manipulation of host immune responses (de Souza and Barrias 2020; Jnana et al. 2023; Retana Moreira et al. 2022). EVs secreted by certain species of bacteria consist of numerous virulence factors including pathogen-associated molecular patterns (PAMPs) such as adhesins that can act as ligands in interaction with host cells (Jnana et al. 2023). In general, EVs help microorganisms interact with their environment, increasing their ability to survive under stress conditions, but specifically, in the infectious process, they contribute to the release of virulence factors which favors its adaptation and survival in hostile environments (Schwechheimer and Kuehn 2015).

The first reports of vesicle release by protozoan parasites date back to 1912 (Schepilewsky 1912) where “appendages” were observed in *Trypanosoma brucei* Bruce, 1895. Later, da Silveira et al. (1979) demonstrated the secretion of plasma membrane vesicles by epimastigote of *Trypanosoma cruzi* Chagas, 1909 (da Silveira et al. 1979) while Silverman et al. (2010) reported the presence of exosomes in *Leishmania donovani* Laveran and Mesnil, 1903 (Silverman et al. 2010). Subsequently, a variety of reports demonstrated the secretion of EVs with other parasitic protozoans. For example, in *Leishmania* spp., differential centrifugation isolated a fraction containing exosomes with a diameter of 30 to 70 nm and exosomal markers such as Hsp70, Hsp90, and elongation factor-1α. Liquid chromatography-tandem mass spectrometry identified more than 400 proteins, of which 52% were orthologs of mammalian exosomal proteins. Furthermore, it was suggested that *Leishmania* spp. use exosomes to communicate with infected and neighboring uninfected macrophages. It is mentioned that the interaction of macrophages with exosomes produced the secretion of IL-8 in a dose-dependent manner. This finding indicated that exosomes could modify specific cytokine profiles of macrophages (Silverman et al. 2010). In the case of *T. cruzi*, it has been suggested that EVs may be responsible for parasite-parasite and parasite-host cell transmission of cellular components (proteins, RNA) through paracrine and/or juxtacrine signaling (de Pablos Torró et al. 2018). Furthermore, label-free quantitative proteomic analysis of EVs from the intracellular parasite *T. cruzi* revealed a rich collection of proteins involved in metabolism, signaling, parasite survival, and virulence. Interestingly, this collection includes proteins related to host-parasite interaction such as surface glycoprotein GP90 and surface protease GP63, proteins related to proteolysis such as calpain cysteine peptidase and cruzipain, and heat shock proteins or chaperones such as Hsp70 (Bautista-López et al. 2017; Bayer-Santos et al. 2013).

In *N. fowleri*, extracellular vesicles have already been analyzed in different contexts. Specifically, it has been observed that EVs can be internalized by macrophages and that these in turn are induced to express costimulatory molecules (CD 80, CD 86, HLA-DR, and CD169) as well as the production of IL-8 (Lertjuthaporn et al. 2022). Furthermore, *N. fowleri* EVs have been shown to be internalized by glial and microglial cells without causing direct cell death, suggesting their involvement in modulating host cell functions. Furthermore, EVs induced an increase in TNF-α, IL-1α, IL-1β, IL-6, IL-17, IFN-γ, MIP-1α, and MIP-2 in microglial cells; these through MyD88-dependent TLR-2/TLR-4 and through NF-κB-dependent MAPK and JAK-STAT signaling pathways (Lê et al. 2023). In addition, in a recent study, EVs from clinical isolates of *N. fowleri* were analyzed for their size, protein profile and proteolytic activity. In this work, a preliminary proteomic profile was reported which includes at least 184 proteins as part of the vesicle content. In addition, the proteolytic activity evaluated with the use of inhibitors revealed the predominance of serine proteases (Retana Moreira et al. 2022). The above, in addition to giving a general overview of the EVs cargo, supports the idea that these can participate in modulating the host immune response, thereby promoting the pathogenesis of this amoeba.

Aside from proteins, extracellular vesicles (EVs) can also contain and transport metabolites, DNA, mRNA, microRNA, and lipids (Lertjuthaporn et al. 2022). It has been reported that exosomal mRNA and microRNA can be transferred from one cell to another, where they remain fully functional in the recipient cell. As a result, they may play crucial roles in cell-to-cell communication (van Niel et al. 2006).

The presence of certain key proteins in extracellular vesicles, such as those considered virulence factors, as well as the presence of RNA molecules containing genetic information that can interfere with transcription processes, suggests that vesicles may play an important role in the biology of protozoa.

The study of specific proteins as part of the EVs cargo released by *N. fowleri* can provide valuable information; this information is not only for a better understanding of the pathogenicity mechanisms used by this amoeba but also to identify biomarkers for timely diagnosis, therapeutic targets, or proteins that can be used for designing vaccines to help prevent, diagnose, or treat PAM. Therefore, the aim of this work was to analyze virulence factors as part of the EVs cargo secreted by *N. fowleri*.

## Methodology

### Amoebic culture

*N. fowleri* ATCC 30808 trophozoites were cultured under axenic conditions at 37 °C in 2% Bacto™ Casitone medium (Gifco) supplemented with 10% fetal bovine serum (FBS) (Gibco) and 0.1% antibiotic–antimycotic (Thermo Scientific). Trophozoites were cooled and collected in the logarithmic phase of growth (48 h).

### Isolation of extracellular vesicles (EVs)

Obtaining EVs from *N. fowleri* trophozoites was done according to what was reported by Retana et al. (2022) with some modifications. Briefly, amoebae were washed three times with sterile PBS and incubated for 24 h at 37 °C in 10 mL of 2% Bacto™ Casitone medium. Subsequently, the supernatants were recovered and centrifuged twice at 2500 × *g* for 15 min at 4 °C to discard cell remnants and then once more at 17,000 × *g* for 30 min at 4 °C to remove large vesicles. The resulting supernatants were recovered, filtered through 0.22-μm pore membrane and concentrated with Centrifugal filters 10 k Amicon^R^ Ultra with centrifugation cycles at 3000 × *g* for 10 min and then ultracentrifuged at 120,000 × *g* for 120 min. The resulting pellet was washed once with filtered PBS at 120,000 × *g* for 120 min and finally resuspended in 100 μL of PBS or 1 mL of TRIzol^R^ Reagent (Invitrogen). Samples were analyzed immediately or stored at − 20 °C until use, no more than 1 week per experiment. The protein concentration was quantified using the Bradford technique.

### Antibodies

For the present study, the antibody selection was as follows: rabbit serum immunized with total extract of *N. fowleri* as anti-*N. fowleri*, rabbit serum immunized with a synthetic peptide of Naeglerioporo B as anti-NPB (unpublished data), rabbit serum immunized with the electroeluted 19 kDa band as anti-19 kDa (Gutiérrez-Sánchez et al. 2023), rabbit serum immunized with the synthetic peptide Smp145 designed from the membrane protein Mp2CL5 as anti-Mp2CL5 (Gutiérrez-Sánchez et al. 2023), antibodies purified from the serum of rabbit immunized with a synthetic peptide of the protease cathepsin B as anti-CatB (Rodríguez-Mera et al. 2022), and mouse anti-β actin (Santa Cruz). Particularly, mouse anti-Hsp70 (Invitrogen) and mouse anti-CD63 exosome (Invitrogen) were used as exosomes markers since they have been the most reported in protozoa (Li et al. 2018; Silverman et al. 2010; Wowk et al. 2017) and they are among the most identified proteins in proteomic studies (Mathivanan et al. 2010). All these mentioned antibodies were used as primary antibodies. On the other hand, donkey anti-rabbit IgG Alexa Fluor 647 or donkey anti-mouse IgG Alexa Fluor 488 for fluorescence and H&L goat anti-rabbit IgG or goat anti-mouse IgG conjugated to horseradish peroxidase for Western blot were used as secondary antibodies.

### Immunocytochemistry

*N. fowleri* trophozoites were incubated on coverslips inside a 24-well plate for 4 h in PBS at 37 °C. Samples were then fixed with 2% paraformaldehyde (PFA) (Sigma-Aldrich) for 40 min and blocked with 1% bovine serum albumin (BSA) (Research Organics) for 30 min at 37 °C.

For immunodetection, slides were incubated overnight with anti-*N. fowleri* or anti-Hsp70, anti-NPB, anti-19 kDa, anti-Mp2CL5, anti-Cathepsin B (1:100 in PBS) as primary antibodies and Alexa Fluor 647 or Alexa Fluor 488 (1:1000 in PBS) as secondary antibodies. All samples were washed three times with PBS after each treatment and incubated with 4,6-diamidino-2-phenylindole (DAPI) for DNA staining. Finally, the samples were mounted with VECTASHIELD (Vector Laboratories, Inc.). Images were taken and analyzed with a fluorescence confocal microscope (Carl Zeiss).

### Protein pattern of extracellular vesicles

The protein profile of EVs was evaluated by SDS-PAGE. Briefly, 5 μg of EVs or 20 μg of total extract was diluted 1:1 in 2 × loading buffer and heated for 5 min at 98 °C and then loaded onto 10% polyacrylamide gels. Electrophoretic runs were performed for 90 min (120 V), and proteins were finally visualized by Coomassie blue R-250.

### Western blot

EVs proteins were separated by SDS-PAGE (10%) as described above and analyzed by Western blot. Briefly, proteins separated on a polyacrylamide gel were transferred to a nitrocellulose membrane. The membrane was blocked with 10% skim milk and incubated at 4 °C for 24 h. Then, samples were incubated with anti-*N. fowleri*, anti-CD63, anti-HSP70, anti-NPB, anti-19 kDa, anti-Mp2CL5, anti-Cathepsin B, or anti-β actin (1:100 in PBS). After 24 h at 4 °C, samples were incubated with anti-rabbit IgG or anti-mouse IgG (1:1000 in PBS) for 24 h at 4 °C. The protein recognition pattern was revealed by adding the substrate (4-chloro naphthol/methanol/H2 O2). The reaction was stopped with phosphate-buffered saline plus 0.005% of Tween-20 (PBS-T).

### mRNA isolation and PCR

Total RNA was isolated from *N. fowleri* trophozoites and EVs using TRIzol™ Reagent (Invitrogen) according to the manufacturer’s protocol. Then, to obtain cDNA, the RevertAid First Strand cDNA Synthesis Kit (Thermo Scientific, K1622) was used according to the manufacturer’s protocol.

To carry out the amplification of the genes, four pairs of oligonucleotides were used to amplify the genes. The Nfa1 primer sequence had been previously reported by Shin et al. (2001) (Shin et al. 2001), while the primers for NPB, Mp2CL5, and CatB were designed using the NCBI-Primer BLAST tool (Ye et al. 2012) and Primer3 (Kõressaar et al. 2018). These primers were all designed from the previously reported sequences: AF154047.1 (NPB), AY049749.1 (Mp2CL5), and KJ159026.1 (CatB) (Herbst et al. 2002; Lee et al. 2014; Réveiller et al. 2001). Primer sequences for each gene are shown in Table 1.

**Table 1:** PCR primers sequence for specific targeted gene

Sequence			
Gen	Forward	Reverse	Tm °C
Nfa1	5′-TGGCCACTACTATTCCAT-3′	5′-AGCACTCCCTTGTACTCC-3′	50
NPB	5′-GCAATGATACCATCATGACCTTGGA-3′	5′-GTCTGTAAGGCTATTGGTGTCTGCTC-3′	57
Mp2CL5	5′-GCCACTGGTATTGCTCTTCC-3′	5′-ATGGGGTAGAGGCAGGTCACT-3′	55
CatB	5′-TGTCACCACAAGATTTGGTATCT-3′	5′-CTGTTTGCACAGGACCGTTG-3′	55

Therefore, polymerase chain reaction (PCR) was performed with the primers (Table 1) and with the DreamTaq Green PCR Master Mix (2x) (Thermo Scientific, K1081). Briefly, amplification reactions were performed in a final volume of 25 μL as described below: 12.5 μL of Dream Taq, 0.5 μL of primer forward, 0.5 μL of primer reverse, 1 μL of cDNA, and 10.5 μL of DNAse-free water. Once the mix had been prepared, samples were placed in a FastGene Ultra Cycler Gradient (FG-TC01) and run under the following PCR conditions: initial denaturation of 1 min at 94 °C and a second of 30 s at the same temperature followed by 40 cycles of amplification and an annealing with 50–61 °C gradient for 30 s, followed by a first 1-min elongation at 72 °C and a final 10-min elongation at 72 °C.

Finally, 5 μL of the resulting products were analyzed by electrophoresis on 0.8% agarose gel (SIGMA) stained with 3 μL of Midori Green Advanced (NIPPON GENETICS, MG04) in 1 × TAE buffer (Cleaver Scientific Ltd). The gel was then displayed on a FastGene® UV transilluminator (FG-300).

## Results

### Extracellular vesicles secretion by *Naegleria fowleri*

**Fig. 1:**
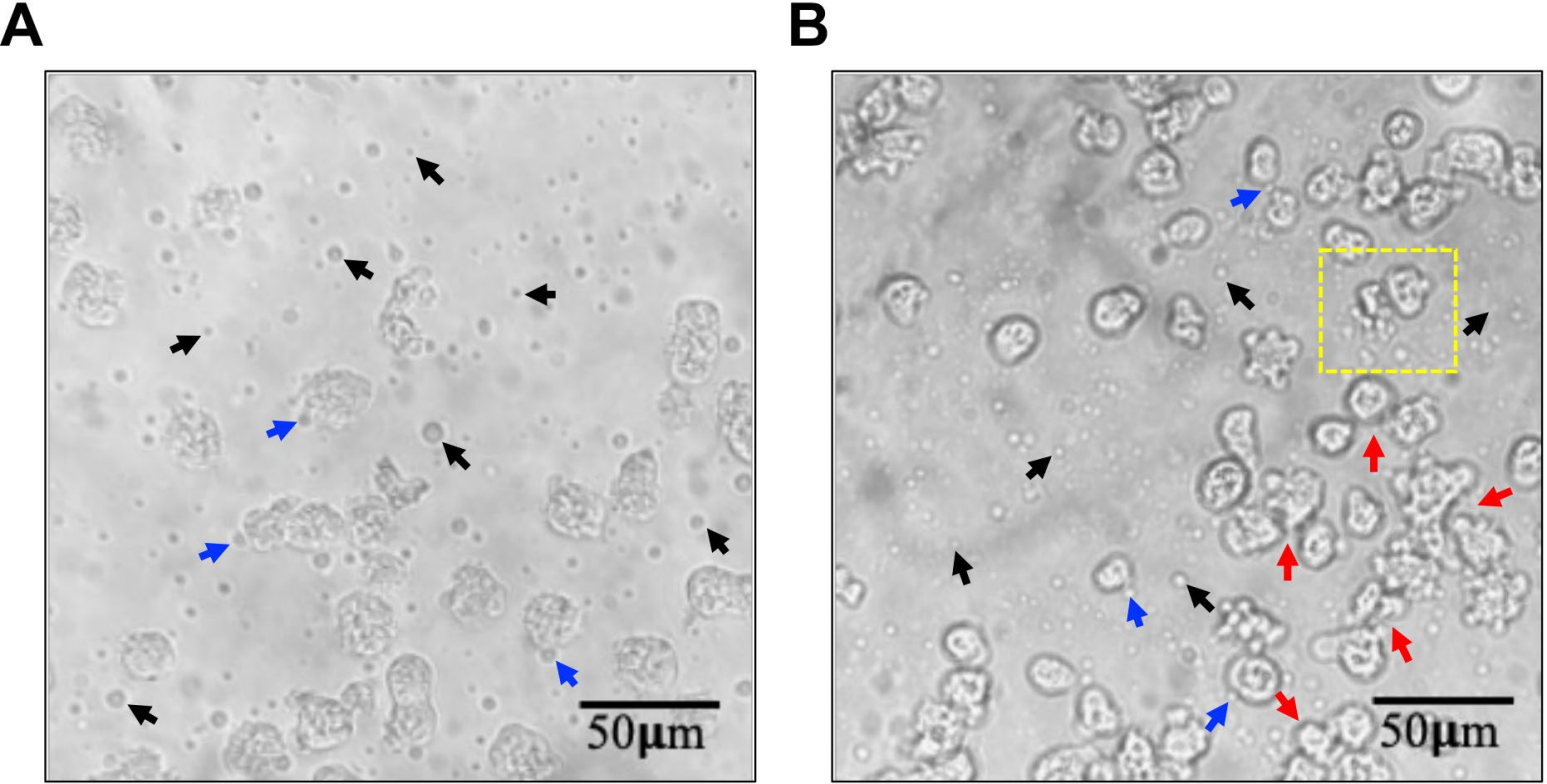
Extracellular vesicles secretion by *Naegleria fowleri*. *N. fowleri* trophozoites observed by light microscopy. **A**, **B** A large variety of vesicles were observed. EVs in extracellular environment (black arrows). EVs in contact with trophozoites (blue arrows). Trophozoites agglutination (red arrows). Vesicles cluster (yellow dotted box). × 40 magnification and scale bar 50 μm

To evaluate vesicle secretion after incubation of trophozoites in PBS for 4 h, trophozoites were observed by light microscopy (Fig. 1). Interestingly, after incubation, we observed small, medium, and large vesicles either released into the extracellular environment (black arrows) or in contact with trophozoites (blue arrows), probably in an exocytosis process (Fig. 1A, B). In addition, we show two fields with different contrasts in which we observed the exocytosis of a wide variety of vesicles of different sizes (Fig. 1A), while on the other, the apparent agglutination of trophozoites and a vesicle cluster being released into the extracellular medium (Fig. 1B, red arrows and yellow dotted box, respectively).

### Immunodetection of *Naegleria fowleri* extracellular vesicles

**Fig. 2:**
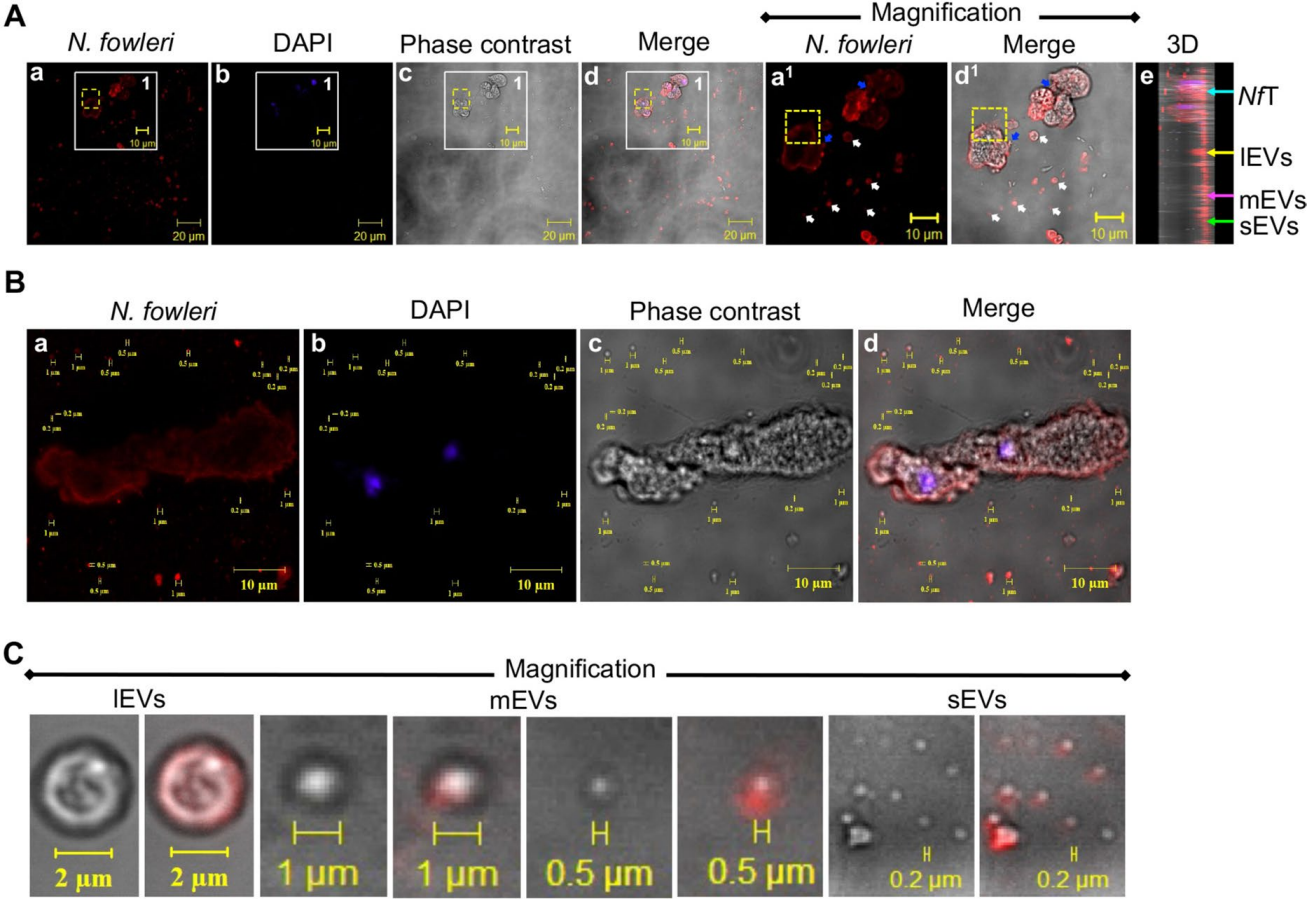
Immunodetection of *Naegleria fowleri* extracellular vesicles. *N. fowleri* trophozoites were maintained in PBS and fixed. **A** Recognitions of trophozoites as well as EVs of *N. fowleri* by anti-*N. fowleri* antibody (red stain). This recognition was observed in a homogeneous manner mainly in the membrane of trophozoites and EVs. Magnified areas (white boxes). Vesicles being released (dotted yellow box). EVs in contact with trophozoites (blue arrows). EVs in extracellular medium (white arrows). 3D images show the side view from magnified area: *Nf*T, *N. fowleri* trophozoites (turquoise arrow). lEVs, large vesicles (yellow arrow). mEVs, medium vesicles (pink arrow). sEVs, small vesicles (green arrow). **B**, **C** Range of vesicles sizes secreted by *N. fowleri*. A range from 0.2 to > 2 μm was identified and all vesicle sizes visualized showed a well-defined spherical shape with an apparent double membrane. lEVs, > 2 μm. mEVs, 0.5–1 μm. sEVs, 0.2 μm or less. DNA is shown in blue stain, and in grayscale, the phase contrast for better visualization of trophozoites and EVs

Once we determined that the incubation conditions stimulated the secretion of a wide variety of EVs, we analyzed their recognition by anti-*N. fowleri* antibody and some morphological characteristics such as size and shape by fluorescence immunocytochemistry (Fig. 2). This analysis showed that both trophozoites and EVs of *N. fowleri* were recognized by anti-*N. fowleri* antibodies (Fig. 2 red staining) and this recognition was distributed in a homogeneous manner mainly in membrane (Fig. 2A, a^1^ and d^1^). In addition, we also identified what appears to be a cluster of vesicles being released into the extracellular medium (Fig. 2A, dotted yellow box), which is consistent with what is shown in Fig. 1B. On the other hand, EVs of different sizes were also observed in contact with trophozoites or in the extracellular medium (Fig. 2A, a^1^ d^1^ blue and white arrows, respectively). In a 3D image, we show a lateral view in which the recognition of trophozoites (white arrow), large (yellow arrow), medium (pink arrow), and small (green arrow) vesicles by anti-*N. fowleri* antibodies is shown (Fig. 2A, e). In addition, we selected a field to clarify the range of vesicles sizes secreted after PBS incubation (Fig. 2B) where we identified vesicles with a range from 0.2 to > 2 μm (Fig. 2B, a, c, and d). Furthermore, all vesicle sizes visualized showed a well-defined spherical shape with an apparent bilayer, and those were recognized by anti-*N. fowleri* antibodies in a well-defined manner in large vesicle membrane or in a less defined manner in medium and small vesicles (Fig. 2C, magnification).

### Immunodetection of virulence factors in *Naegleria fowleri* extracellular vesicles

**Fig. 3:**
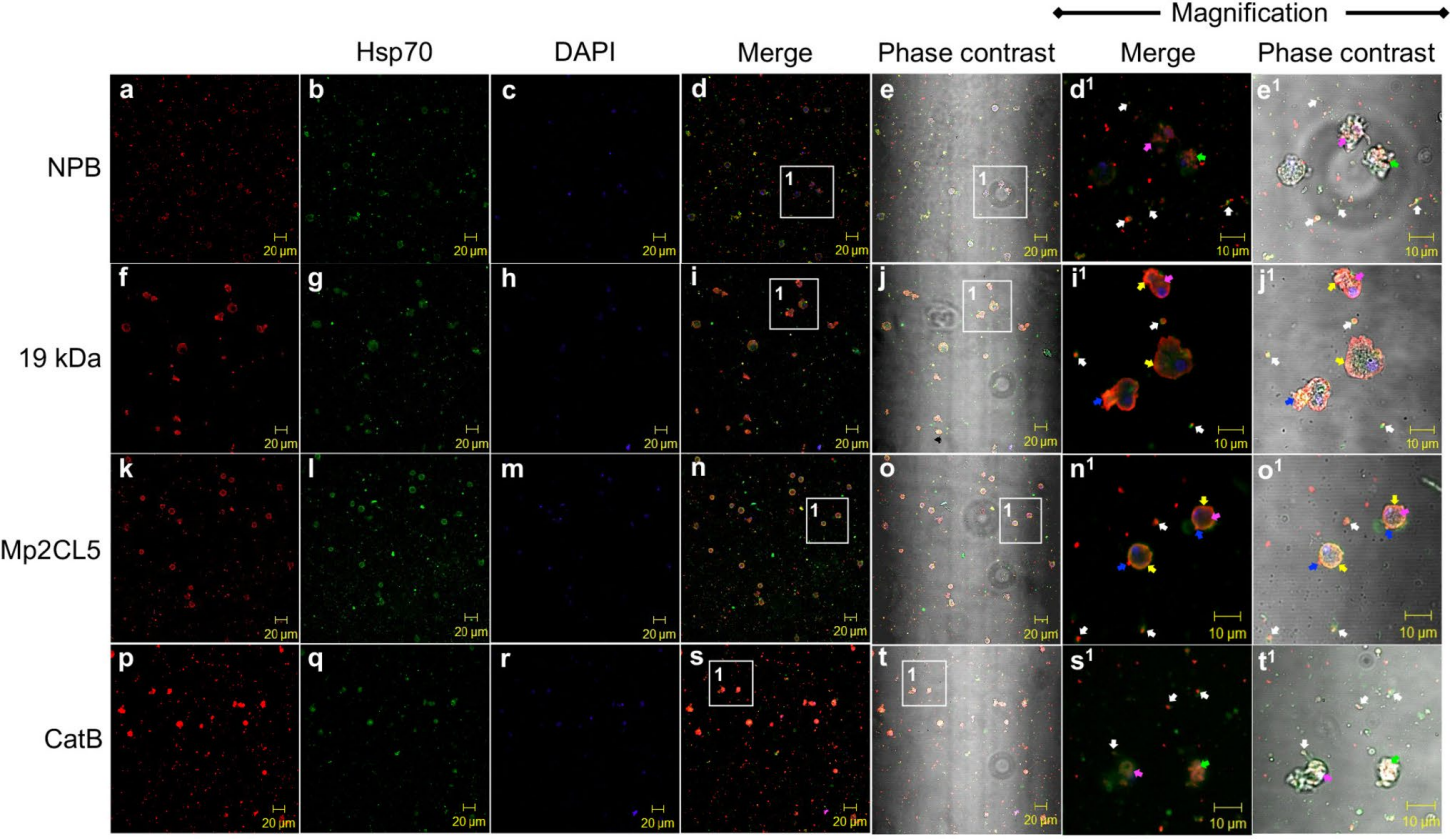
Immunodetection of virulence factors in *Naegleria fowleri* extracellular vesicles. *N. fowleri* trophozoites were maintained in PBS and fixed. Recognitions of trophozoites as well as EVs of *N. fowleri* by anti-NPB (**a**–**e**), anti-19 kDa (**f**–**j**), anti-Mp2CL5 (**k**–**o**), anti-CatB (**p**–**t**) red stain, and as an EVs marker anti-Hsp70 (**b**, **g**, **l**, and **q**, green stain). This recognition was observed mainly in the trophozoites membrane (yellow arrows), cytoplasm (pink arrows), food-cups (blue arrows), and vesicles (green arrows) as well as part of the EVs cargo (white arrows). DNA is shown in blue stain, and in grayscale, the phase contrast for better visualization of trophozoites and EVs. Magnified representative areas (white boxes)

Once the recognition of EVs by anti-*N. fowleri* antibodies was evaluated, we decided to determine if part of the EVs cargo contains any virulence factor of *N. fowleri* such as NPB, 19 kDa, Mp2CL5, and CatB, using specific antibodies to these factors. It is important to mention that anti-Hsp70 antibody was used as EVs marker. Interestingly, trophozoites as well as EVs were recognized by all the above-mentioned antibodies with some differences in their distribution (Fig. 3, red and green staining). In the case of trophozoites, NPB and CatB were observed mainly in the cytoplasm either homogeneously distributed or in small vesicles (Fig. 3d, d^1^ and s, s^1^ pink and green arrows, respectively) while 19 kDa band and Mp2CL5 were predominantly observed in the membrane with a homogeneous distribution or in food-cups or vesicles (Fig. 3i, i^1^ and n, n^1^ yellow and blue arrows, respectively). Regarding EVs, all proteins were apparently localized as part of the content of both large and small vesicles or distributed in the membranes (Fig. 3, white arrows). Specifically, NPB, Mp2CL5, and CatB were predominantly localized in EVs (Fig. 3e, e^1^, o, o^1^, and t, t^1^ white arrows), while 19 kDa band was also observed inside and in the membrane (Fig. 3j, j^1^ white arrows). It is important to mention that some vesicles showed positive staining for anti-virulence factor antibodies and other vesicles were only positive for Hsp70. In addition, we also observed vesicles without staining.

### Protein pattern of *Naegleria fowleri* extracellular vesicles

**Fig. 4:**
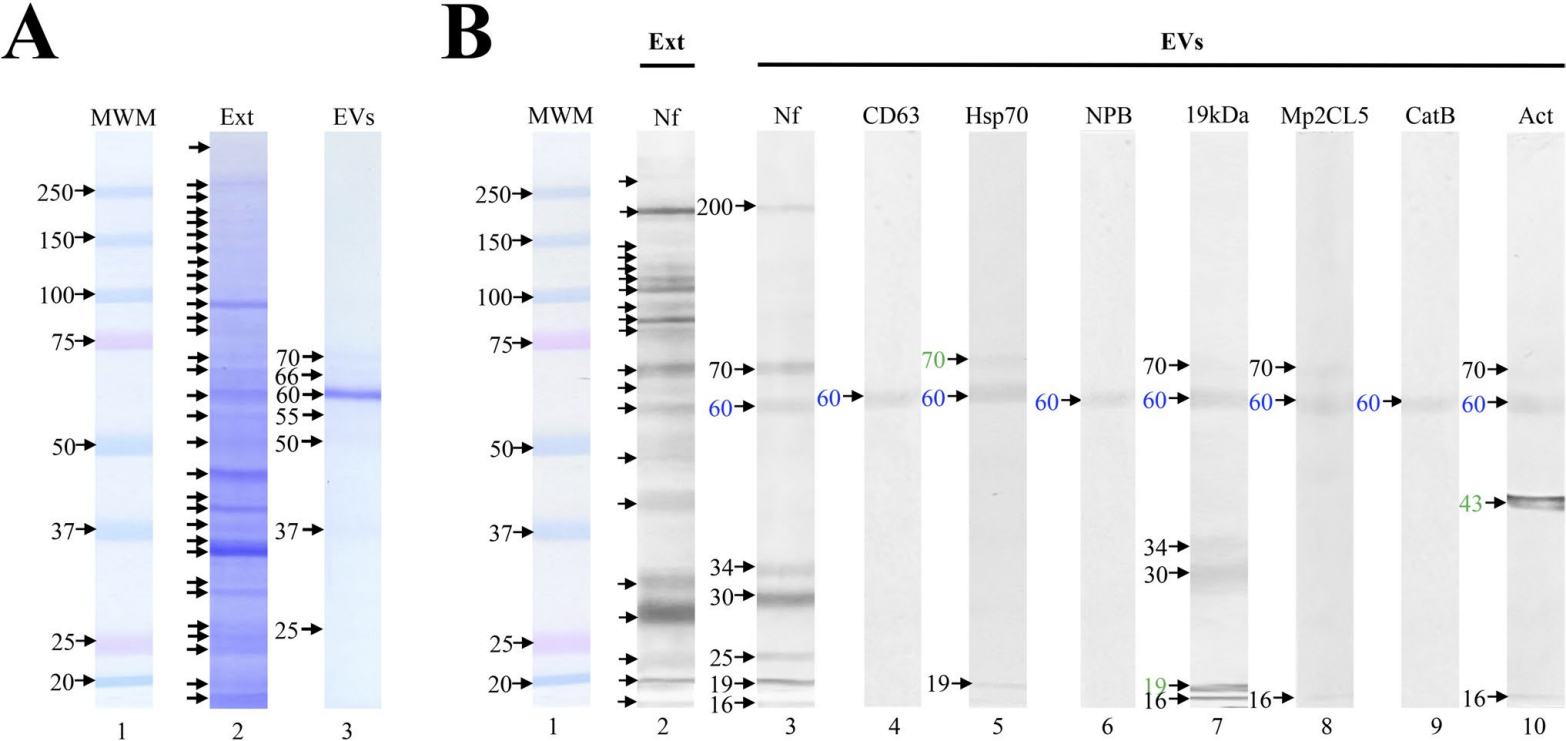
Protein pattern of *Naegleria fowleri* extracellular vesicles. Ten micrograms of total extract (Ext) of *N. fowleri* as well as 5 µg of extracellular vesicles (EVs) purified from culture medium diluted 1:1 with 2 × buffer were analyzed by SDS-PAGE and Western blot. **A** Polyacrylamide gel stained with Coomassie blue. The recognition pattern for ET was from 250 to 17 kDa (Line 2) while for EVs was from 70 to 25 kDa (Line 3). **B** Nitrocellulose membrane. The recognition pattern for ET incubated with anti-N. fowleri was 250 to 17 kDa (Line 2), while in the EVs incubated with the same antibody, the bands from 200 to 17 kDa were recognized (Line 3). CD63 (line 4) and HSP70 (line 5) were used as controls which showed recognition of bands of 60 kDa (CD63) and 70, 60, and 19 kDa (HSP70). Anti-NPB recognized only the 60 kDa band (Line 6), anti-19 kDa recognized bands with a range of 70 to 17 kDa (Line 7), anti-Mp2CL5 recognized the bands of 70, 60, and 17 kDa (Line 8), anti-CatB recognized only the 60 kDa band (Line 9), and anti-β actin recognized the 70, 60, 43, and 17 kDa bands (Line 10). Polypeptide recognized by all antibodies (blue numbers). Molecular weight reported for each protein (green numbers)

Using immunocytochemistry, we observed that NPB, 19 kDa band, Mp2CL5, and CatB proteins can be released in EVs; however, to verify this, we decided to evaluate the proteins pattern present in EVs of culture media by means of the immunoblot technique using the same antibodies than in immunocytochemistry (Fig. 4). First, the proteins of the total extracts of *N. fowleri* or EVs were separated in polyacrylamide gels by SDS-PAGE and stained with Coomassie blue. We observed in total extract of *N. fowleri* polypeptide bands in a range of 270 to 17 kDa, while in the EVs, only seven bans were observed with a molecular weight of 70, 66, 60, 55, 50, 37, and 25 kDa (Fig. 4A, lines 2 and 3, respectively). After, other polyacrylamide gel was transferred to nitrocellulose membranes and incubated with anti-*N. fowleri* (as a control), NPB, 19 kDa band, Mp2CL5, CatB, or actin antibodies. The recognition pattern of the total extract incubated with anti-*N. fowleri* antibodies includes polypeptides of low and high molecular weight with a range from 270 to 17 kDa (Fig. 4B, line 2). This is similar to what was observed in the Coomassie blue gel. However, unlike what was observed in the gel, the recognition pattern observed in the EVs incubated with anti-*N. fowleri* antibody includes polypeptides of both high and low molecular weight with a range of 200 to 17 kDa (Fig. 4B, line 3). In this case, unlike in immunocytochemistry, in addition to anti-Hsp70, we used anti-CD63 as an EV marker. Interestingly, both markers recognized polypeptides present in EVs; in the case of anti-CD63, only the recognition of the 60 kDa polypeptide was observed (Fig. 4B, line 4), while with anti-Hsp70, the recognition of the 70, 60, and 19 kDa polypeptides was observed, although 70 kDa is the molecular weight estimated by this antibody (Fig. 4B, line 5). As for anti-NPB, it only recognized the 60 kDa polypeptide (Fig. 4B, lane 6), while anti-19 kDa band recognized the 70, 66, 60, 34, 25, 19, and 17 kDa polypeptides, since the antibodies against this band recognize different peptides contained in this band (Fig. 4B, lane 7), anti-Mp2CL5 recognized the 70, 60, and 17 kDa polypeptides (Fig. 4B, lane 8), while anti-CatB only recognized the 60 kDa polypeptide (Fig. 4B, lane 9), and anti-actin recognized the 70, 60, 43, and 17 kDa polypeptides, being 43 kDa, the estimated weight for the antibody used (Fig. 4B, lane 10).

### Molecular content of *Naegleria fowleri* extracellular vesicles

**Fig. 5:**
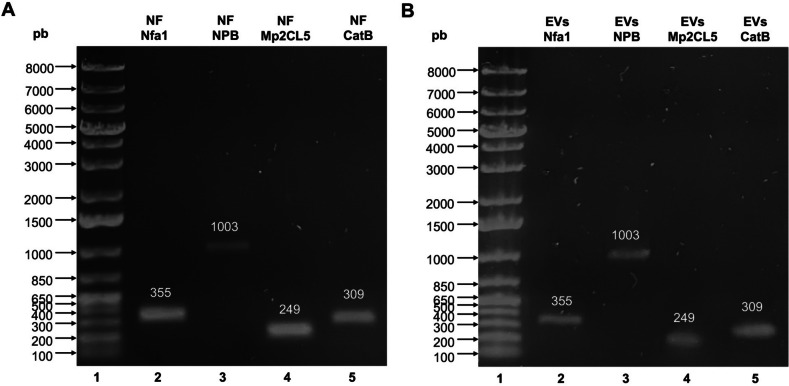
PCR products of *Naegleria fowleri* trophozoites and extracellular vesicles. Midori Green stained agarose gel (0.8%) showing the size of PCR product of *N. fowleri* trophozoites and EVs. **A** PCR products of *N. fowleri* trophozoites. **B** PCR products from *N. fowleri* EVs. In both, line 1, molecular size marker, line 2, Nfa1 (355 pb), line 3, NPB (1003 pb), line 4, Mp2CL5 (249 pb), and line 5, CatB (309 pb)

At this point, we know that EV content can include the proteins NPB, 19 kDa band, Mp2CL5, CatB, and actin while CD63 and Hsp70 can also be included in the membranes; however, it has been widely described that in addition to proteins, the EV content can include genetic material, so we decided to evaluate the presence of mRNA for the genes Nfa1, NPB, Mp2CL5, and CatB by means of RT-PCR method (Fig. 5). Interestingly, in both cases, for trophozoites (Fig. 5A) and EVs (Fig. 5B), PCR products showed amplification for the four genes evaluated, obtaining the expected weights: 355 bp for Nfa1 (Fig. 5A, B, Line 2), 1003 bp for NPB (Fig. 5A, B, Line 3), 249 bp for Mp2CL5 (Fig. 5A, B, Line 4), and 309 bp for CatB (Fig. 5A, B, Line 5).

## Discussion

In recent years, evidence of EVs secretion as well as their characterization and composition has become a topic of interest in many research groups since it has been described that these vesicles can fulfill different functions. In general, it has been reported that extracellular vesicles can transfer bioactive components such as nucleic acids and proteins from donor to recipient cells, thus facilitating the exchange of information between cells, and play a crucial role in interacting with the environment, enhancing their ability to survive under stress conditions, releasing virulence factors, and modulating the immune system during the infectious process (Milane et al. 2015). These functions aid in their adaptation and survival in hostile environments (de Souza & Barrias 2020; Gavinho et al. 2018; Schwechheimer and Kuehn 2015). Furthermore, we recently suggested that cathepsin B and its mRNA were being released in small vesicles following the interaction of *N. fowleri* trophozoites with polymorphonuclear or in the nasal cavity of infected mice (Rodríguez-Mera et al. 2022). Therefore, we decided to assess the morphological characteristics of vesicles secreted by *N. fowleri* trophozoites and determine whether the vesicle cargo includes proteins considered as important virulence factors.

Interestingly, a wide variety of vesicle sizes were observed using either light or confocal microscopy (Figs. 1 and 2, respectively). According to MISEV2018, EVs can be divided into medium/large EVs (> 200 nm) and small EVs (< 200 nm) based on their physical properties (Théry et al. 2018), but based on their origin, EVs can also be classified into apoptotic bodies (50–1000 nm), microvesicles formed from outward budding and pinching of the plasma membrane (100–1000 nm), and exosomes derived from endocytotic multivesicular bodies (30–150 nm) (Jia et al. 2022; Milane et al. 2015). The above suggests that based on size, incubation of *N. fowleri* trophozoites in PBS induced mainly not only the secretion of exosomes and microvesicles but also a small number of apoptotic bodies. This is supported by the fact that we observed what appear to be multivesicular bodies releasing their contents into the extracellular medium, suggesting that the EVs observed are not only of ectosomal origin but also of endosomal (Fig. 2A). Regarding their morphology, all EVs exhibited a spherical shape and a bilayer resembling the structure reported in various protozoa (de Souza and Barrias 2020).

Regarding EVs cargo, we evaluated specific proteins that are considered important virulence factors for *N. fowleri* trophozoites (Figs. 3 and 4, respectively). For this, we used Hsp70 and CD63 as exosome markers since it has been widely reported that exosomes, compared to other EVs, have specific markers such as CD9, CD63, CD81, HSP70, and HSP90 (Liu et al. 2022; Shtam et al. 2020), being Hsp70 and CD63 the most reported in protozoa (Li et al. 2018; Silverman et al. 2010; Wowk et al. 2017). Furthermore, these proteins are also highly represented in proteomic analysis of exosomes from various cell types (Mathivanan et al. 2010).

In our experiments, EVs were recognized by both anti-Hsp70 and anti-CD63 antibodies. However, the recognition of EVs as well as trophozoites with anti-Hsp70 antibody was interesting since this antibody not only detected vesicles distributed in the cytoplasm of trophozoites and those that were secreted but also recognized trophozoites surface and structures known as food-cups. Flores-Suárez et al. (2024) suggest that the presence of Hsp70 on the trophozoites surface may be related to adhesion contributing to *N. fowleri* invasion and migration to the brain. On the other hand, the most important function of HSPs is to act as molecular chaperones (Ohtsuka and Suzuki 2000), suggesting that Hsp70 could also be translocating proteins to propel them across the membrane; however, further studies are needed to verify this.

As we mentioned above, the EVs analyzed in this work were recognized not only either by Hsp70 or CD63 but also by NPB, 19 kDa polypeptide band, Mp2CL5, cathepsin B, and β-actin proteins. These proteins were selected for analysis as part of the EV cargo since they have been attributed a role in the pathogenesis caused by *N. fowleri*, either because they are overexpressed in highly virulent strains of *N. fowleri*, because of their cytopathic and cytotoxic effect, or because they are distributed in the cytoplasm, membrane, or in cytoplasmic extensions such as food-cups or pseudopod-like structures (Young and Lowrey 1989) (Gutiérrez-Sánchez et al. 2020; Réveiller et al. 2001) (Herbst et al. 2002) (Zysset-Burri et al. 2014) (Sohn et al. 2019) (Gutiérrez-Sánchez et al. 2023) (Lee et al. 2014) (Seong et al. 2017) (Rodríguez-Mera et al. 2022). In addition, some are considered antigenic and immunogenic proteins (Gutiérrez-Sánchez et al. 2020) (Rojas-Hernández et al. 2020). Interestingly, all the antibodies used recognized the 60 kDa polypeptide band, which suggests the presence of peptide sequences from each of the proteins evaluated as part of the cargo of the EVs secreted by *N. fowleri*. In addition, the fact that, in some cases, not only the 60 kDa polypeptide was recognized by some of the antibodies used suggests the presence of more than one isoform of the proteins evaluated.

The concept that EVs represent a form of intercellular communication is based on the idea that a wide variety of cells release a cargo compartmentalized with proteins, lipids, and nucleic acids for uptake and integration into other cells (Gavinho et al. 2018). For example, label-free quantitative proteomic analysis of EVs from the intracellular parasite *T. cruzi* revealed a rich collection of proteins involved in metabolism, signaling, parasite survival, and virulence. Interestingly, this collection includes proteins related to host-parasite interaction such as surface glycoprotein GP90 and surface protease GP63 and proteins related to proteolysis such as calpain cysteine peptidase and cruzipain (Bautista-López et al. 2017; Bayer-Santos et al. 2013). Interestingly, cruzipain is the main cysteine protease of *T. cruzi* which has been implicated in the parasites metabolism and is considered an important candidate for vaccine development and for the design of trypanocidal drugs (Duschak and Couto 2009). On the other hand, the protease GP63 has been previously suggested as potential candidates for diagnosis of *Trypanosoma rangeli* Uribe, 1929 infections (Wagner et al. 2013). Regarding *N. fowleri*, Retana et al. (2022) characterized and identified components of the protein cargo of EVs from clinical isolates of *N. fowleri*. They report a preliminary proteomic analysis which revealed the presence of 184 proteins including actin, Hsp70, and some proteases (Retana Moreira et al. 2022). In addition, we observed that the main distribution of the evaluated proteins was in the membrane of both trophozoites and EVs. This indicates that the membrane may be a key location for the amoeba to exert its pathogenic potential. These findings highlight the potential of these proteins as candidates for vaccine development or as biomarkers for early diagnostic tests.

Finally, we used PCR analysis to determine the mRNA content specific for selected virulence factors present in EVs isolated from *N. fowleri* culture supernatants. Surprisingly, among the mRNAs evaluated in the EVs, we found Nfa1, NPB, Mp2CL5, and cathepsin B. The relevance of the data obtained lies in the fact that these EVs were recovered from trophozoite maintained in culture medium and not from trophozoites subjected to some kind of stimulus such as interaction with a cell line or trophozoites recovered from the brain, where they would be expected to produce significant amounts of mRNA that could be pathologically relevant. In a previous study, it was reported that infection of fibroblasts with *Toxoplasma gondii* Nicolle and Manceaux, 1908 triggers the production of exosome-like vesicles containing 4 mRNA species with neurological activities including Rab-13, eukaryotic translation elongation factor 1 alpha 1, thymosin beta 4, and LLP homolog, which are suggested to alter brain chemistry to the point of producing behavioral changes, making the exact location of the cells take a backseat as the effect is spread throughout the brain by EVs secretion from infected cells (Pope and Lässer 2013). Furthermore, it has been reported that exosomal mRNAs and microRNAs transferred from one cell to another are fully functional in the recipient cell and may therefore play key roles in cell-to-cell communication (van Niel et al. 2006). For example, incubation of human cells with exosomes from mouse cells led to the synthesis of mouse proteins from the mRNAs present in the exosomes, indicating horizontal gene transfer (Izquierdo-Useros et al. 2011; Lakkaraju and Rodriguez-Boulan 2008).

## Conclusion

Although the role that these vesicles play in either the communication or the infectious process caused by *N. fowleri* is not described in this document, we can assume that the virulence factors detected in this work as part of the EV cargo either as protein or as mRNA could play a role in pathogenesis since the proteins evaluated have been implicated as part of the arsenal that *N. fowleri* uses to cause damage to the host.

This study presents evidence supporting a model in which exosome release may serve as one of the primary mechanisms for protein and mRNA secretion. These may contribute to *N. fowleri* ability to survive in harsh environments and affect the adhesion and invasion process. However, further research is required to fully understand the intricate interactions between *N. fowleri* and its environment. This understanding will help in the development of therapeutic treatments, vaccine design, and timely diagnosis of infections caused by these protozoa.

## Supplementary Information

Below is the link to the electronic supplementary material.

Supplementary file1 (DOCX 616 KB)

## Data Availability

No datasets were generated or analyzed during the current study.
