# Supplementary material for: Analysis of virulence factors in extracellular vesicles secreted by Naegleria fowleri
Source: Parasitol Res. 2024 Oct 21;123(10):357. doi: 10.1007/s00436-024-08378-9 (PMC11493829; doi:10.1007/s00436-024-08378-9)
Supplement: Supplementary file 1 — Supplementary file1 (DOCX 616 KB) [file 436_2024_8378_MOESM1_ESM.docx]

**Figure 1. Polyacrylamide gel stained with Coomassie blue**


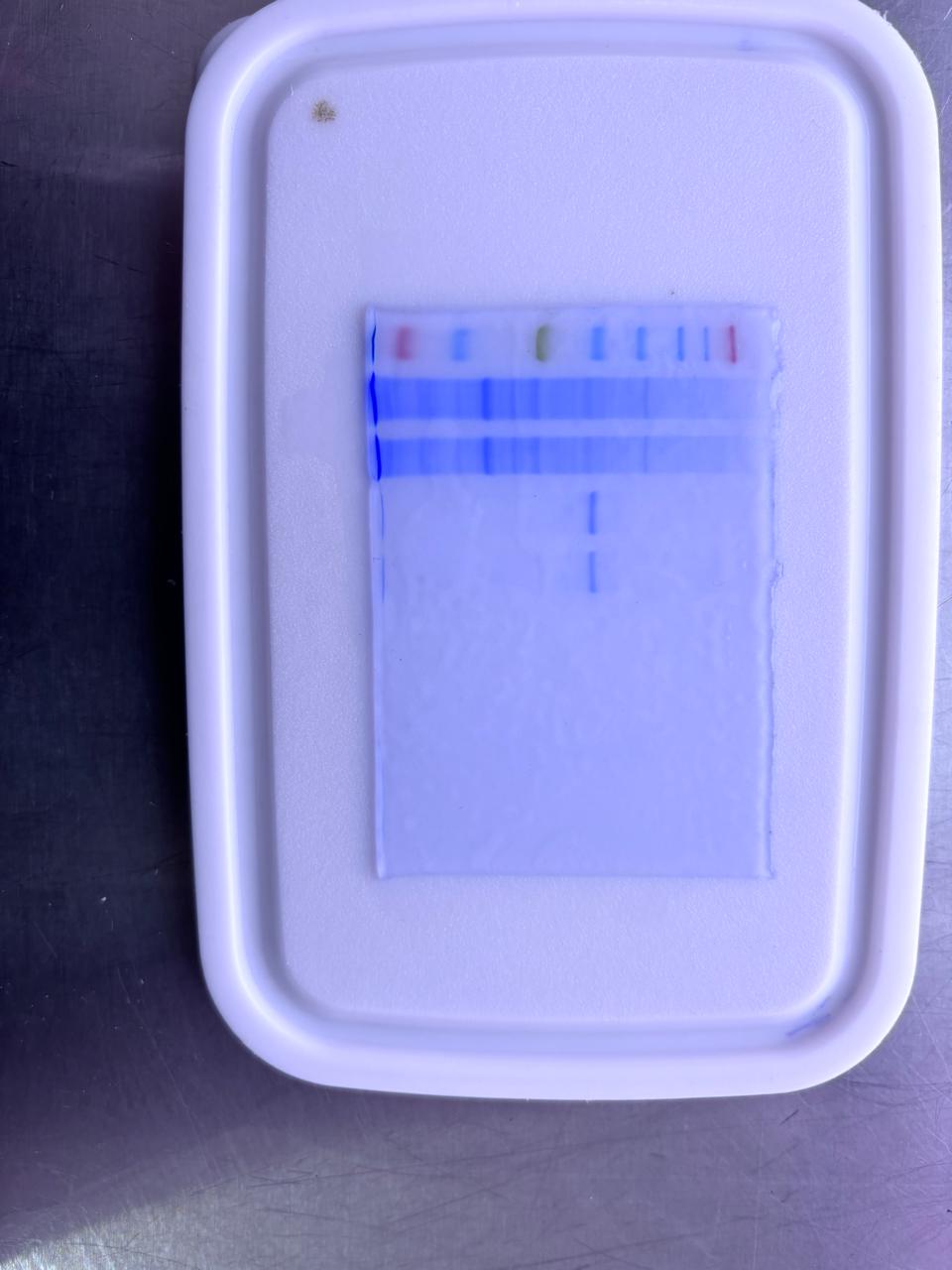


**Figure 1.** **Protein pattern of *Naegleria fowleri* extracellular vesicles.** 10 µg of Total Extract (ET) of *N. fowleri* as well as 5 µg of extracellular vesicles (EVs) purified from culture medium diluted 1:1 with 2x buffer were analyzed by SDS-PAGE. Polyacrylamide gel stained with Coomassie blue. The recognition pattern for ET was from 250 to 17 kDa (Line 2 and 3) while for EVs was from 70 to 25 kDa (Line 4 and 5).

**Figure 2. Nitrocellulose membrane for Western blot analysis**


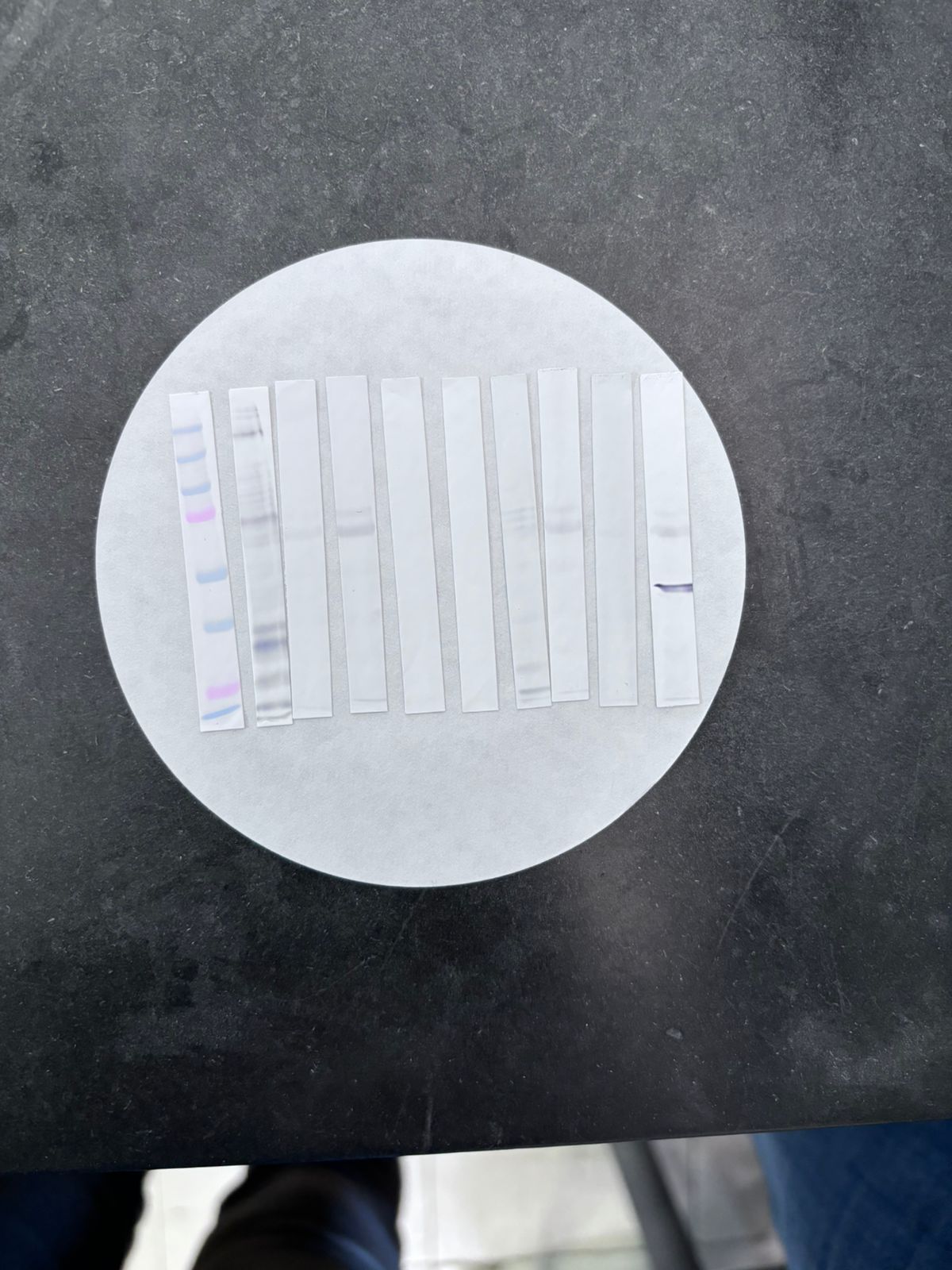


**Figure 2.** **Protein pattern of *Naegleria fowleri* extracellular vesicles.** 5 µg of extracellular vesicles (EVs) purified from culture medium diluted 1:1 with 2x buffer were analyzed by Western blot. Nitrocellulose membrane. The recognition pattern for EVs incubated with anti-N. fowleri was 200 to 17 kDa (Line 2). CD63 (line 3) and HSP70 (line 4) were used as controls which showed recognition of bands of 60 kDa (CD63) and 70, 60 and 19 kDa (HSP70). Anti-NPA recognized only the 60 kDa band (Line 5, unused data), anti-NPB recognized only the 60 kDa band (Line 6) anti-19 kDa recognized bands with a range of 70 to 17 kDa (Line 7), anti-Mp2CL5 recognized the bands of 70, 60 and 17 kDa (Line 8), anti-CatB recognized only the 60 kDa band (Line 9) and anti-β actin recognized the 70, 60, 43 and 17 kDa bands (Line 10).

**Figure 3. Agarose gel with PCR products of *N. fowleri* trophozoites.**


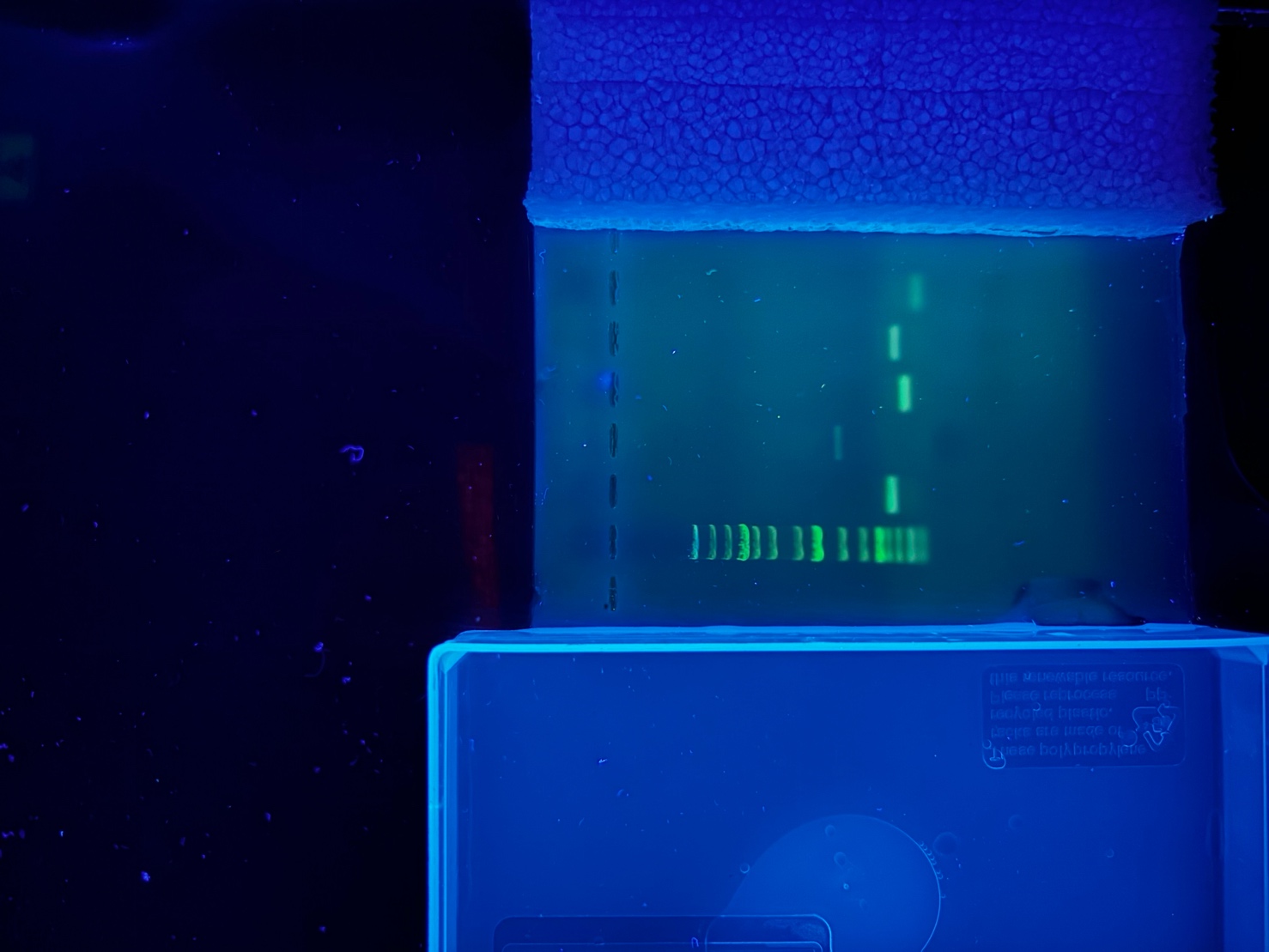


**Figure 3. PCR products of *Naegleria fowleri* trophozoites.** Midori Green stained agarose gel (0.8%) with PCR product of *N. fowleri* trophozoites. Line 1, molecular size marker, line 2, Nfa1 (355 pb), line 3, NPB (1003 pb), line 4, Mp2CL5 (249 pb) and line 5, CatB (309 pb).

**Figure 4. Agarose gel with PCR products of *N. fowleri* extracellular vesicles.**


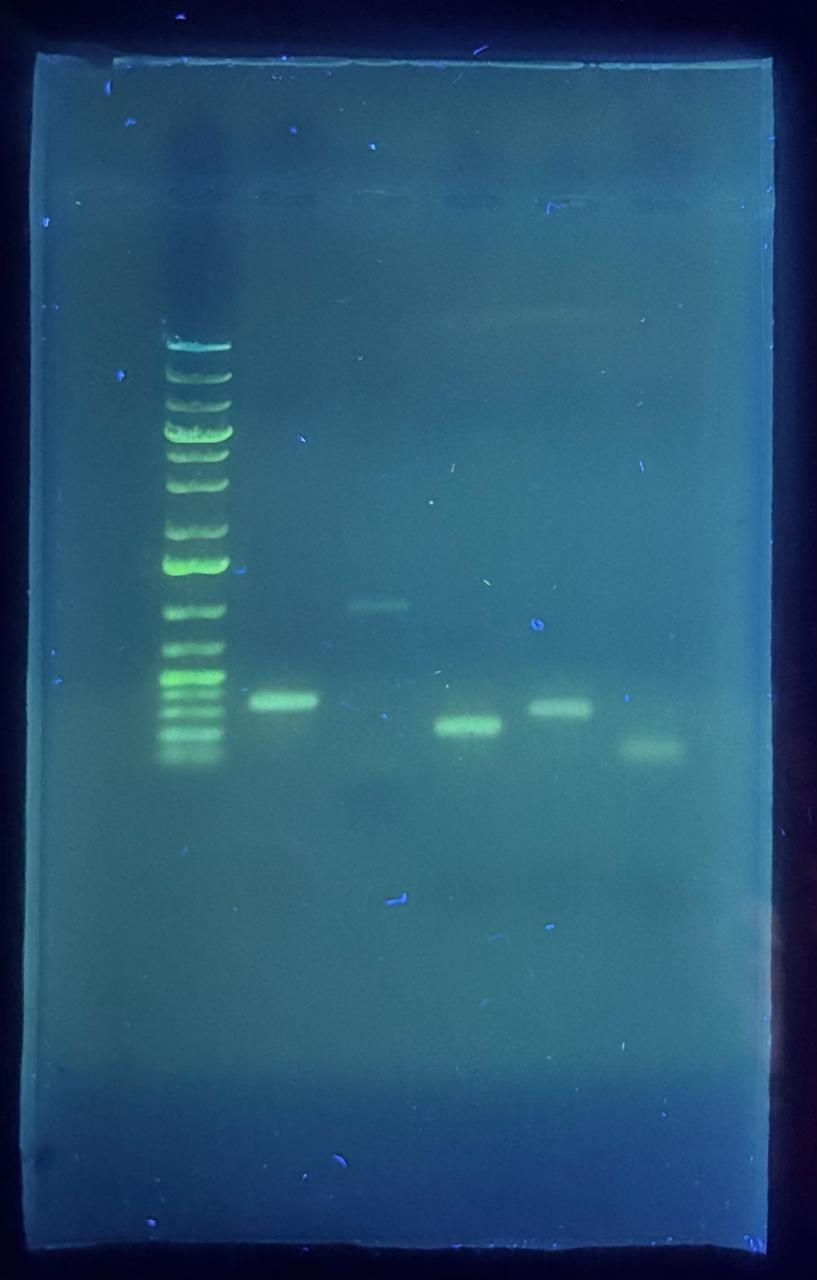


**Figure 4. PCR products of *Naegleria fowleri* extracellular vesicles.** Midori Green stained agarose gel (0.8%) with PCR product of *N. fowleri* EVs. Line 1, molecular size marker, line 2, Nfa1 (355 pb), line 3, NPB (1003 pb), line 4, Mp2CL5 (249 pb) and line 5, CatB (309 pb).
